# The sphingosine-1-phosphate pathway is differentially activated in human gestational tissues

**DOI:** 10.1210/jendso/bvag074

**Published:** 2026-03-28

**Authors:** Magdaleena Naemi Mbadhi, Hideji Fujiwara, Ruth Gill, Kaci T Mitchum, Zixi (Cici) Lin, Nandini Raghuraman, Antonina I Frolova

**Affiliations:** Center for Reproductive Health Sciences, Department of Obstetrics and Gynecology, Washington University School of Medicine, St. Louis, MO 63110, USA; Division of Endocrinology, Metabolism & Lipid Research, Washington University School of Medicine, St. Louis, MO 63110, USA; Center for Reproductive Health Sciences, Department of Obstetrics and Gynecology, Washington University School of Medicine, St. Louis, MO 63110, USA; Center for Reproductive Health Sciences, Department of Obstetrics and Gynecology, Washington University School of Medicine, St. Louis, MO 63110, USA; Center for Reproductive Health Sciences, Department of Obstetrics and Gynecology, Washington University School of Medicine, St. Louis, MO 63110, USA; Division of Maternal Fetal Medicine, Department of Obstetrics and Gynecology, Washington University School of Medicine, St. Louis, MO 63110, USA; Center for Reproductive Health Sciences, Department of Obstetrics and Gynecology, Washington University School of Medicine, St. Louis, MO 63110, USA; Division of Maternal Fetal Medicine, Department of Obstetrics and Gynecology, Washington University School of Medicine, St. Louis, MO 63110, USA

**Keywords:** decidua parietalis, metabolomics, myometrium, S1P, sphingolipids, labor

## Abstract

**Background:**

Dysregulated myometrial contractility contributes to obstetric complications. Sphingosine-1-phosphate (S1P), a bioactive lipid, modulates inflammation and smooth muscle contractility, including uterine contractility. However, its metabolic dynamics during pregnancy are poorly characterized. This study profiled S1P metabolic enzymes and receptors, and quantified sphingolipid metabolites in human gestational tissues across pregnancy.

**Methods:**

Myometrium, decidua parietalis, and chorioamnion were collected from women undergoing cesarean sections at term (≥37 weeks’ gestation) without labor (TNL, *n*  *=* 8), term with labor (TL, *n*  *=* 5), and preterm (<37 weeks’ gestation) without labor (PTNL, *n*  *=* 6). Messenger RNA (mRNA) expression of S1P metabolic enzymes and receptors was assessed using quantitative polymerase chain reaction, while sphingolipids were quantified using targeted liquid chromatography-tandem mass spectrometry.

**Results:**

S1P metabolic enzymes and receptors were differentially expressed across gestational tissues. At TNL, sphingosine kinase1 (*SPHK1*) expression was higher in the decidua parietalis than in the chorioamnion and myometrium. The myometrium exhibited the highest mRNA expression of S1P receptors (*S1PR1-4*) compared to the decidua and chorioamnion. At term, S1P was more abundant in the myometrium than in the decidua parietalis and chorioamnion. Both *SPHK1* and S1P were higher in TL than in TNL myometrium. S1P levels were higher in the myometrium at TNL than at PTNL, with no significant differences in the decidua or chorioamnion. Overall, sphingolipid metabolism was highest in the decidua and myometrium and lowest in the chorioamnion at term.

**Conclusion:**

These findings reveal tissue-specific regulation of S1P metabolism and signaling in human gestational tissues, suggesting S1P's therapeutic role to modulate myometrial contractility.

Parturition involves three key phenomena: the degradation of fetal membranes, synchronous myometrial contractions, and cervical ripening and dilation [1, 2]. The physiological mechanisms governing human pregnancy and parturition are crucial for ensuring healthy pregnancy and labor outcomes. Preterm labor results from a premature activation of labor-inducing factors, triggering early myometrial contractility [3, 4]. Conversely, prolonged gestation (delivery after 42 weeks) and labor arrest result from delayed onset or inadequate activation of labor-inducing factors, leading to insufficient myometrial contraction [5, 6]. These labor abnormalities significantly increase the risk of neonatal morbidity and mortality and often necessitate delivery by cesarean section, which increases maternal morbidity rates [7-10]. To mitigate these labor complications and improve maternal and neonatal outcomes, we need to better understand the underlying mechanisms governing parturition.

The bioactive lipid mediator sphingosine-1-phosphate (S1P) was identified as a potential mediator of parturition, as it upregulated the expression of inflammatory markers associated with parturition in human myometrial cells [11]. S1P is generated by phosphorylation of sphingosine by the sphingosine kinase enzymes, SPHK1 and SPHK2 [12]. SPHK1 is primarily located in the cytoplasmic compartment and translocates to the plasma membrane, where it is the principal generator of extracellular S1P [13]. In contrast, SPHK2 is located mainly in the cell nucleus, endoplasmic reticulum, and mitochondria and produces intracellular S1P that regulates diverse intracellular functions such as gene transcription, mitochondrial respiration, and apoptosis [14-16]. Once exported from the cell, S1P mediates its effects in an autocrine or paracrine fashion by binding and activating one of its five G protein-coupled receptors, S1P receptor 1-5 (S1PR1-5). S1P can then be recycled back to sphingosine by S1P phosphatases, S1PP1 and S1PP2, or irreversibly broken down by S1P lyase (S1PL) into non-sphingoid molecules [17].

Accumulating evidence supports the involvement of S1P metabolic enzymes in pregnancy maintenance and parturition onset. For example, *Sphk1*-deficient mice exhibited increased decidual cell death, uterine hemorrhage, and decreased trophoblast cell invasion, resulting in impaired decidualization and pregnancy loss [18]. SPHK1 activity and expression increase with advancing gestation in human decidua [19], and its expression is higher in the myometrium during term labor than before labor onset [11]. Additionally, global inhibition of SPHK activity has been shown to inhibit lipopolysaccharide-induced preterm birth in mice [20, 21]. Taken together, these studies suggest a potential physiological role for S1P in human parturition. However, the primary site of S1P production in the uterus has not been defined. We hypothesized that S1P synthesis is initiated in the decidua, triggering a signaling cascade that activates S1P-related enzymes and receptors in the myometrium to promote labor onset.

Here, we quantified the expression of enzymes involved in S1P synthesis and metabolism, as well as S1P receptors, in the fetal (chorioamniotic) membranes, decidua parietalis, and myometrium from women at term before or during labor. Furthermore, we used targeted mass spectrometry to quantify the concentrations of sphingolipid metabolites, including S1P, in these tissues. Finally, we compared the abundance of S1P and its two major precursors, sphinganine and sphingosine, in gestational tissues at preterm and at term pregnancy.

## Materials and methods

### Study population

This is a cross-sectional study of nulliparous women who presented for delivery and underwent cesarean section at a single tertiary care institution between August 2022 and January 2024. Washington University in St. Louis Human Research Protection Office approved this study (IRB ID# 202205099). Multiple gestations and women with known HIV, hepatitis C or hepatitis B infections and gestational age ≥42 weeks were excluded.

Patients presenting for scheduled cesarean section at term (≥37 weeks of gestation) were assigned to the term non-labor group (TNL, *n*  *=* 8), while those undergoing cesarean delivery in labor were assigned to the term labor group (TL, *n*  *=* 5). Patients undergoing unlabored cesarean section prior to 37 weeks of gestation were assigned to the preterm non-labor group (PTNL, *n*  *=* 6). Patient clinical characteristics, including indications for cesarean delivery, are presented in Table 1.

**Table 1:** Patient characteristics

Characteristics	Term pregnancy		Preterm pregnancy	
	TNL (*n* *=* 8)	TL (*n* *=* 5)	PTNL (*n* *=* 6)	*P*-value
Maternal age (years)	35.4 ± 5.1	31.4 ± 5.9	33.0 ± 6.7	.49
Gestational age (weeks)	38.6 ± 1.0	38.9 ± 1.4	34.0 ± 1.7	<.001 (0.62 for TNL vs TL)
Nulliparous	3 (37.5)	4 (80)	0	.02 (0.13 for TNL vs TL)
Length of labor (hours)	N/A	12.0 ± 7.3	N/A	N/A
Indication for c/section:				.047 (0.39 for TNL vs PTNL)
Malpresentation	3 (37.5)	0	1 (16.7)	
Arrest of labor disorder	0	3 (60)	0	
Maternal indication/complications	0	1 (20)	0	
Non-reassuring fetal status	0	0	0	
Repeat c/section	4 (50.0)	1 (20)	5 (83.3)	
Other^*a*^	1 (12.5)	0	0	
BMI at delivery (kg/m^2^)	31.3	36.0	35.9^*b*^	.30
Prepregnancy diabetes mellitus	1 (12.5)	1 (20.0)	0	.55
Gestational diabetes mellitus	0	0	2 (33.3)	.09
Chronic hypertension	0	0	2 (33.3)	.09
Hypertensive disorder of pregnancy	3 (37.5)	4 (80.0)	4 (66.7)	.28
Tobacco use during pregnancy	0	0	0	N/A
Alcohol use during pregnancy	1 (12.5)	0	0	N/A

Data represented are mean ± SD or n (%); BMI, body mass index.

^*a*^Fetal microsomia in in the setting of T1DM.

^*b*^Missing values *n*  *=* 2.

### Sample collection

Immediately after delivery of the neonate and placenta, an approximately 1.5 × 1.0 × 0.5 cm sample of myometrium was collected from the upper portion of the uterine incision site, placed into ice cold physiologic buffer solution (Dulbecco's phosphate buffered saline, pH = 7.4, supplemented with 1% charcoal-stripped fetal bovine serum (Gibco), 25 µg/mL gentamicin, 0.25 µg/mL amphotericin B) in the operating room, and immediately transported to the lab. Once in the lab, the myometrial tissue was cut into ∼50 mg strips. The placenta was collected after delivery and transported to the lab. Decidua parietalis tissue was separated from the fetal membranes as previously described [22]. Briefly, approximately a 10 cm^2^ piece of fetal membrane was cut and spread on a sterile surface with the maternal side facing up. Using a sterile tissue scraper, the decidual tissue was scraped from the underlying fetal membranes. Approximately 50 mg of the collected tissue was then placed in a cryovial. An approximately 50 mg piece of fetal membranes (chorioamnion) remaining following removal of the decidua parietalis was collected. All samples were flash frozen in liquid nitrogen within 30 minutes of collection and stored at −80 °C until further processing and analysis.

### RNA isolation and quantitative polymerase chain reaction

Total RNA was isolated from the fetal membrane, decidua, and myometrium using the Direct-zol RNA Miniprep Kit (Zymo Research). DNase was added to remove any genomic DNA. RNA quality and quantity were assessed using the Nanodrop spectrophotometer. Total RNA (800 ng) was reverse-transcribed to cDNA using a high-capacity cDNA reverse transcription kit (Applied Biosystems) as per manufacturer's protocol. cDNA was prepared, and mRNA gene expression was analyzed using real-time PCR (ABI7500; Life Technologies, MA, USA) using SYBR Green Master Mix as per manufacturer's protocol. Cycling conditions were as follows: denaturation at 95 °C for 10 minutes, followed by 40 cycles of 95 °C for 15 seconds and annealing temperature at 60 °C for 1 minute, with a melt curve from 60-95 °C. Human GAPDH was used as an internal control for gene expression normalization and exhibited comparable Ct values across all samples. Fold changes in gene expression were calculated using the 2^−ΔΔCT^ method. Primers used in this study were designed using PrimerQuest (Integrated DNA Technologies). Primer efficiencies were ≥85%, and specificity was validated using the melt curve and electrophoresis analysis of the amplified product. Sequences of all primer sets used in the study are detailed in Table S1 [23].

### Sphingolipid analysis by liquid chromatography-tandem mass spectrometry (LC-MS/MS)

Four volumes of ice-cold water were added to each tissue (50-140 mg) followed by homogenization in an Omni Bead Blaster (Perkin Elmer Inc) at 4 °C for several minutes. The LC-MS/MS analysis was slightly modified from a previously published protocol [24]. Briefly, Sphingolipids (sphingoid bases, ceramides, and sphingomyelins (SM)) were extracted from 50 µL of the homogenate with 400 µL of (1:1) methanol/isopropyl alcohol containing internal standards (sphingosine-d7 10 ng, sphinganine-d7, 5 ng, S1P-d7 20 ng, Cer(d18:1 17:0) 200 ng, SM (d18: 1 17:0) 200 ng).

For sphingoid bases (sphingosine, sphinganine, and S1P), 10 µL of the extract was injected on a Water Xbridge C18 column (3.5 µm, 4.6 mm × 100 mm; Waters Corp.) connected to Shimadzu 20 ADX HPLC and API-4500 Qtrap tandem mass spectrometer (Applied Biosystem). The HPLC solvents were selected as A: 1% formic acid in water and B: 1% formic acid in 1:1 methanol/ACN. The solvent gradient was used from 70% B to 100% in 4 minutes at a flow rate of 1 mL/min and back to 70% in 2 minutes for the next injection. The positive ion multiple reaction monitoring (MRM) method was used to monitor sphingoid base analytes, including internal standards. Four-point calibration samples (0.8 ng/mL, 8 ng/mL, 20 ng/mL, and 40 ng/mL) were prepared to obtain the absolute quantification data. All sphingoid base samples were injected twice to obtain the average data.

For ceramides and sphingomyelins (SM) ((Cer(d18:1 16:0), Cer(d18:1 18:0), Cer(d18:1 20:0), Cer(d18:1 22:0), Cer(d18:1 24:1), and Cer(d18:1 24:0) SM(d18:1 16:0), SM(d18:1 18:0), SM (d18:1 20:0), SM(d18:1 22:0), SM(d18:1 24:1), and SM(d18: 24:0)) 1 µL of the extract was injected on to an Agilent Zorbax Eclipse Plus C8 column (3.5 µm, 3.0 mm × 100 mm; Agilent) for HPLC/MS/MS analysis. The solvents were selected as A: 10 mM ammonium acetate in 30% ACN in water B: 10 mM ammonium acetate in 1:1 methanol/isopropyl alcohol. The solvent gradient was used from 65% B to 100% in 3 minutes at a flow rate of 1 mL/min and back to 65% in 2 minutes for the next injection. The positive ion MRM method was used for all ceramide and sphingomyelin (SM) detection, including the internal standards. 3- to 4-point calibration samples were also prepared for ceramide and SM absolute quantification. All samples were injected twice for the average data. All sphingolipids, including deuterated sphingolipids, were purchased from Avanti Polar Lipids Inc, 700 Industrial Park Drive. Alabaster, AL 35007. Analyst 5.1 software (Applied Biosystem) was used for this analysis.

### Statistical analysis

Statistical analyses were performed using GraphPad Prism software. The unpaired t-test with Welch correction assessed the statistical significance between two groups. Normality of data distribution was assessed by inspecting Q-Q plots and formally assessed by performing Shapiro–Wilk and Kolmogorov–Smirnov tests within groups, and no strong deviations from normality were detected. For comparisons involving three groups, a one-way ANOVA was utilized, while a two-way ANOVA was applied for more than three groups, using Holm-Sidak's method for multiple comparisons. Data was considered statistically significant at *P value ≤0.05, **P value ≤0.01, and ***P value ≤0.001.

## Results

### SPHK1 expression is highest in the decidua parietalis, and S1P receptor expression is highest in the myometrium at term non-labor

**Figure 1:**
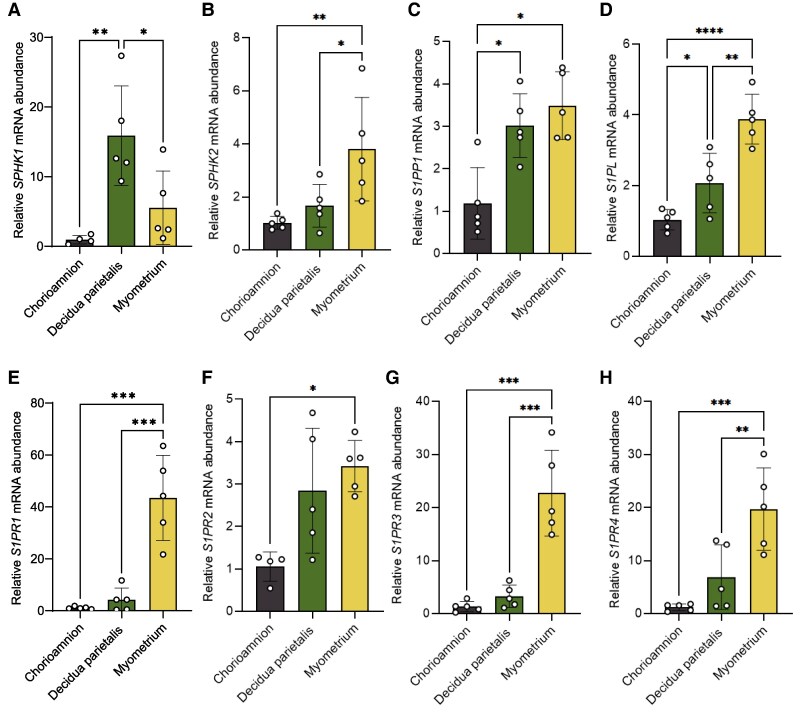
S1P metabolic enzyme and receptor mRNA expression in the human chorioamnion, decidua parietalis, and myometrium at term non-labor (TNL). mRNA expression of (A) sphingosine kinase 1 (SPHK1), (B) sphingosine kinase 2 (SPHK2), (C) sphingosine-1-phosphate phosphatase1 (S1PP1), (D) sphingosine-1-phosphate lyse (S1PL) in the human amnion, decidua parietalis, and myometrium during TNL were analyzed by quantitative real-time PCR (qPCR). Sphingosine-1-phosphate receptors (S1PRs), including (E) S1PR1, (F) S1PR2, (G) S1PR3, and (H) S1PR4 mRNA expressions, were also analyzed by qPCR in the human amnion, decidua parietalis, and myometrium at TNL. Presented are means ± SD of *n*  *=* 5. Statistical significance was determined by using one-way ANOVA and adjusted for multiple comparisons using the Holm-Sidak method (*P < .05; **P < .01; ***P < .001; ****P < .0001).

Previous works have analyzed the expression of S1P metabolic enzymes and receptors at term gestation in the decidua [19] and myometrium [11] independently. To further characterize S1P metabolism during parturition, we examined the expression of S1P-synthesizing enzymes (*SPHK1* and *SPHK2*), degradation enzymes (*S1PP1* and *S1PL*), and receptors (S1PR1-4) in matched chorioamnion, decidua parietalis, and myometrium from patients at TNL. We found that mRNA for all enzymes and receptors analyzed was expressed in the tissues examined (Fig. 1). *SPHK1* mRNA was significantly more abundant in the decidua parietalis than in the chorioamnion and myometrium (Fig. 1A). *SPHK2* mRNA was highest in the myometrium (Fig. 1B). *S1PP1* mRNA was significantly more abundant in the decidua parietalis and myometrium than in the chorioamnion (Fig. 1C). S1PL mRNA levels were lowest in the chorioamnion, increased in the decidua parietalis, and showed the highest expression in the myometrium. (Fig. 1D).

Expression of all four S1P receptors analyzed was highest in the myometrium and lowest in the chorioamnion (Fig. 1E-1H). *S1PR1* mRNA was 40-fold higher in the myometrium than in the chorioamnion and 5-fold higher than in the decidua parietalis (Fig. 1E). *S1PR3* mRNA was 17-fold higher in the myometrium than in the chorioamnion and 6.8-fold higher than in the decidua parietalis (Fig. 1G).

### SPHK1 and S1PR3 are differentially expressed during labor in the human myometrium at term

**Figure 2:**
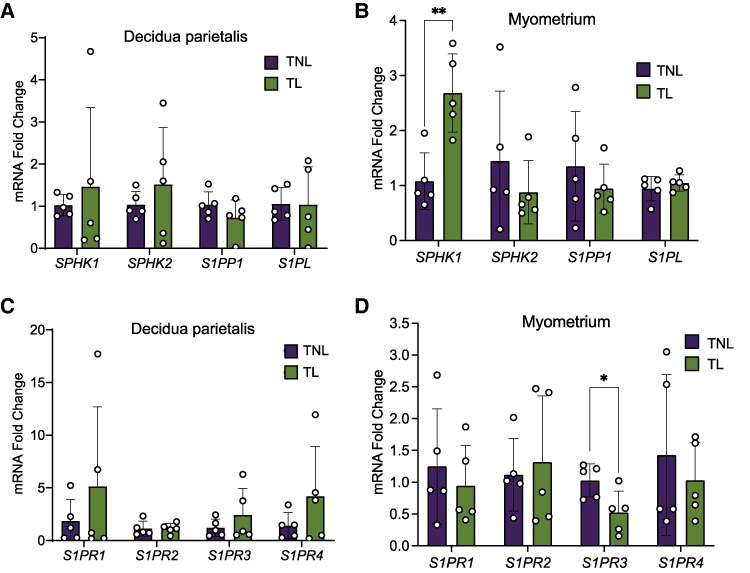
S1P metabolic enzyme and receptor mRNA expression in the human decidua parietalis and myometrium at term non-labor (TNL) and term-labor (TL). Expression of sphingosine kinase1 (SPHK1), sphingosine kinase2 (SPHK2), sphingosine-1-phosphate phosphatase1 (S1PP1), and sphingosine-1-phosphate lyse (S1PL) mRNA in the (A) decidua parietalis and (B) myometrium at TNL (*n*  *=* 5) and TL (*n*  *=* 5) were analyzed by quantitative real-time PCR (qPCR). Expression of sphingosine-1-phosphate receptors (S1PRs), including S1PR1, S1PR2, S1PR3, and S1PR4 mRNA in the (C) decidua parietalis and (D) myometrium at TNL and TL were analyzed by qPCR. Presented are means ± SD. Statistical significance was determined by using the unpaired t-test with Welch correction (*P < .05; **P < .01).

Since S1P metabolic enzymes and receptors were most abundantly expressed in the decidua parietalis and myometrium, we compared their expression in these two tissues between patients at TNL and TL. Expression of IL8 was used as a marker of labor and notably increased in TL myometrial samples (Fig. S1) [23]. In the decidua parietalis, expressions of *SPHK1, SPHK2, S1PP1, S1PL*, and *S1PR1-4* did not differ between TNL and TL (Fig. 2A and 2C). In the myometrium, *SPHK2, S1PP1, S1PL, S1PR1, S1PR2,* and *S1PR4* expression also did not differ between TNL and TL (Fig. 2B and 2D). However, myometrial expression of *SPHK1* was 2.5-fold higher at TL than at TNL (Fig. 2B), whereas *S1PR3* expression was significantly higher at TNL than at TL (Fig. 2D).

### Sphingolipid abundance within gestational tissues at term

**Figure 3:**
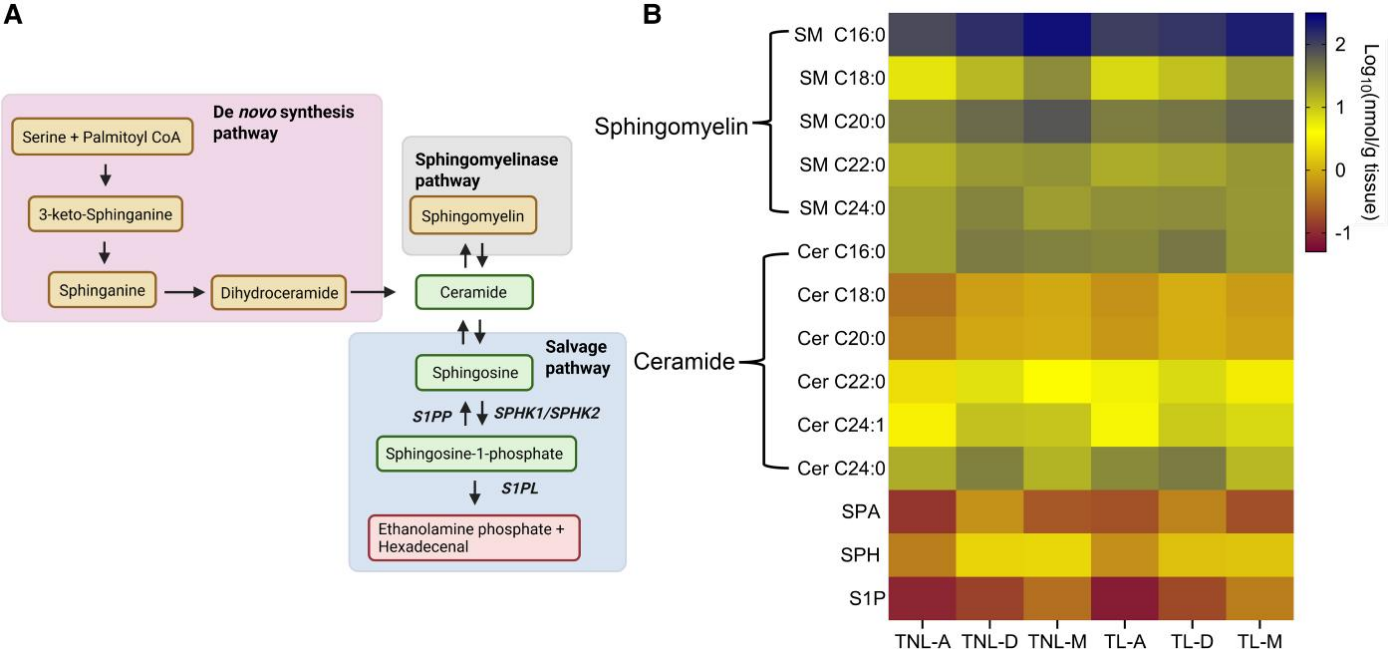
Distribution of sphingolipids in the chorioamnion, decidua parietalis, and myometrium at term non-labor (TNL) and term-labor (TL). (A) Schematic pathway of the sphingolipid synthesis. (B) Heatmap depicting the relative concentrations and distributions of 14 sphingolipids in the chorioamnion, decidua parietalis, and myometrium of TNL (*n*  *=* 8) and TL (*n*  *=* 5) quantified by mass spectrometry (LC-MS/MS). Sphingolipid relative abundance was log-transformed; the blue color bar represents high abundance, the yellow bar represents modest abundance, and the maroon color bar represents low abundance. Abbreviations: A: chorioamnion; D: decidua parietalis; Cer: ceramide; M: myometrium; SM: sphingomyelin; SPA: sphinganine; SPH: sphingosine; S1P: sphingosine-1-phosphate; TL: term labor; TNL: term non-labor.

To further explore the S1P synthesis pathway (Fig. 3A) at term pregnancy, we used LC-MS/MS to quantify the abundance and distribution of 14 targeted sphingolipids in the chorioamnion, decidua parietalis, and myometrium at TNL and TL (Fig. 3B). Consistent with previous reports on sphingolipid abundance in the serum and plasma [25, 26], our results showed that sphingomyelins (SM) were also the most abundant sphingolipids in gestational tissues at term pregnancy. Specifically, SM C16:0 was the most abundant sphingomyelin at both TNL and TL.

Ceramides, followed by sphingosine (SPH)—the direct S1P precursor—were the next most abundant sphingolipids in the chorioamnion and uterine tissues. Among ceramides, the fatty acid subclasses C16:0, C22:0, C24:0, and C24:1 were more abundant than C18:0 and C20:0 in all three tissues at both TNL and TL.

Sphinganine (SPA), the upstream precursor for ceramides and S1P in the *de novo* synthesis pathway, and S1P itself were the least abundant across all tissues. Specific concentrations of all sphingolipids across the different tissues at TNL and TL are provided in Tables S2-S6 [23].

**Figure 4:**
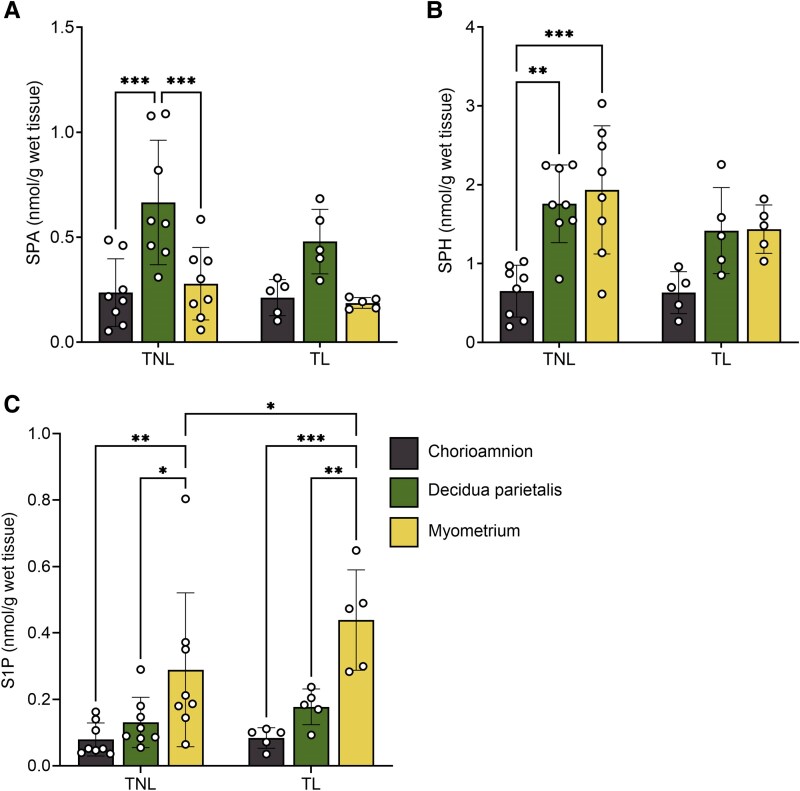
Bioactive sphingolipid concentrations in the human chorioamnion, decidua parietalis, and myometrium at term non-labor (TNL) and term labor (TL). (A) Sphinganine (SPA), (B) Sphingosine (SPH), and (C) sphingosine-1-phosphate (S1P) concentrations in the chorioamnion, decidua parietalis, and myometrium were analyzed by mass spectrometry (LC-MS/MS) in patients at TNL (*n*  *=* 8) and TL (*n*  *=* 5). Presented are means ± SD. Statistical significance was determined by using two-way ANOVA and adjusted for multiple comparisons using the Holm-Sidak method (*P < .05; **P < .01).

To identify the primary site of these bioactive lipid mediators, we compared SPA, ceramide, SPH, and S1P concentrations across the three gestational tissues and labor states. SPA concentrations were higher in the decidua parietalis than in the myometrium and chorioamnion (Fig. 4A). At TNL, ceramide-C16:0, -C18:0, -C20:0, -C22:0, and -C24:1 were higher in the decidua parietalis than in the chorioamnion, with no significant difference compared to the myometrium. At TL, ceramide-C18:0, -C20:0, and -C24:1 levels remained elevated in the decidua parietalis compared to the chorioamnion, and ceramide-C16:0, -C18:0, -C22:0, and -C24:1 were significantly more abundant in the decidua parietalis than in the myometrium (Tables S2 and S3) [23].

Similarly, SPH concentrations were comparable between the decidua parietalis and myometrium but lower in the chorioamnion at TNL, with no notable differences across tissues at TL (Fig. 4B). In contrast, S1P showed a distinct pattern, with the lowest levels in the chorioamnion, higher in the decidua parietalis, and reaching the highest levels in the myometrium at both TNL and TL (Fig. 4C). Additionally, S1P levels were higher in the myometrium at TL compared to TNL (Fig. 4C).

### S1P production is higher in the myometrium at term labor than at term non-labor

**Figure 5:**
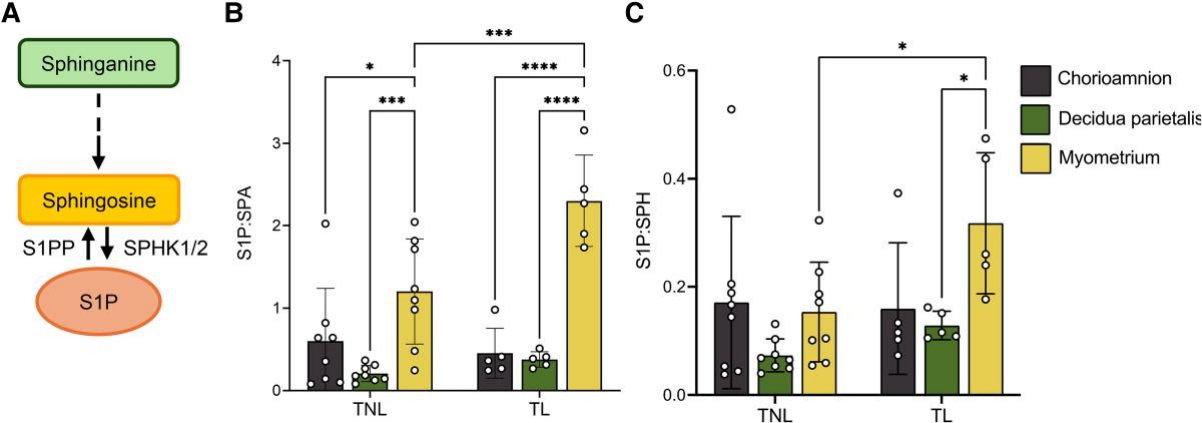
S1P to precursor ratios in the human chorioamnion, decidua parietalis, and myometrium at term non-labor (TNL) and term labor (TL). (A) Schematic pathway of S1P synthesis. (B) S1P-sphinganine (S1P:SPA) and (C) S1P-sphingosine (S1P:SPH) ratio in the human chorioamnion, decidua parietalis, and myometrium at TNL (*n*  *=* 8) and TL (*n*  *=* 5). Presented are means ± SD. Statistical significance was determined by using two-way ANOVA and adjusted for multiple comparisons using the Holm-Sidak method (*P < .05; **P < .01).

Given the differential abundance of S1P and its precursors (SPA and SPH) across gestational tissues, we next analyzed the ratios of S1P to its precursors as a proxy for turnover rate (Fig. 5A). The S1P:SPA ratio was highest in the myometrium compared to the chorioamnion and decidua parietalis at term (Fig. 5B). Additionally, both the S1P:SPA and S1P:SPH ratios were significantly higher in the myometrium at TL than at TNL (Fig. 5B and 5C), suggesting a higher S1P production rate in the myometrium during labor.

### Sphingolipid abundance in preterm gestational tissues

**Figure 6:**
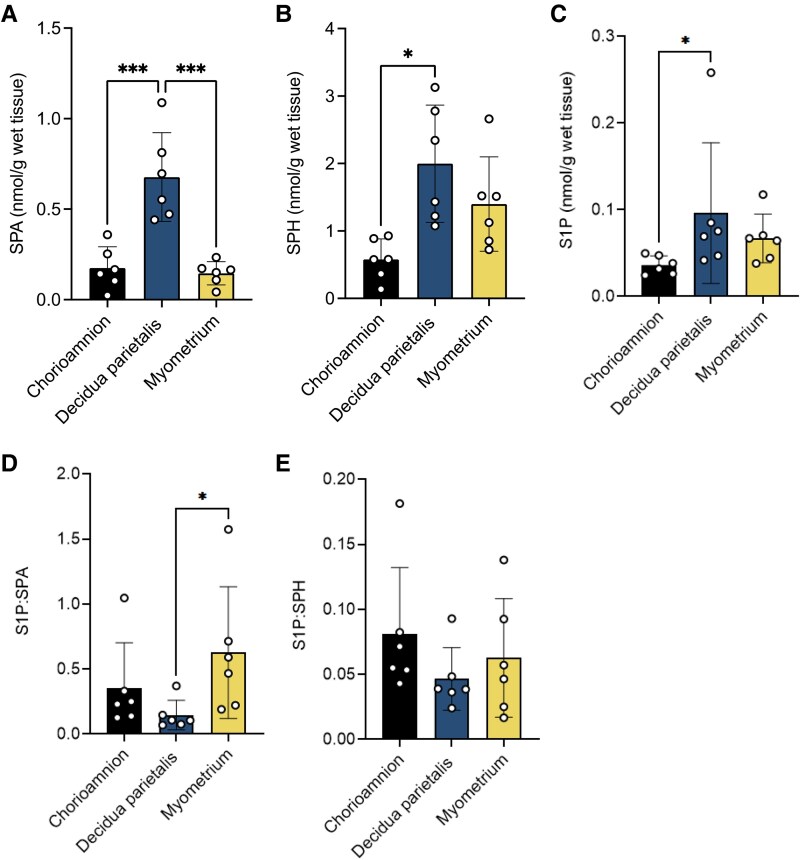
The bioactive sphingolipid concentrations in the human chorioamnion, decidua parietalis, and myometrium at preterm non-labor (PTNL). (A) Sphinganine (SPA), (B) Sphingosine (SPH), and (C) sphingosine-1-phosphate S1P concentrations in the chorioamnion, decidua parietalis, and myometrium were analyzed by mass spectrometry (LC-MS/MS) in patients at PTNL (*n*  *=* 7). (D) The S1P to sphinganine ratio (S1P:SPA) and (E) S1P to sphingosine ratio (S1P:SPH) in the chorioamnion, decidua parietalis, and myometrium was determined at PTNL (*n*  *=* 7). Presented are means ± SD. Statistical significance was determined using one-way ANOVA and adjusted for multiple comparisons using the Holm-Sidak method (*P < .05; **P < .01).

To explore whether similar sphingolipid metabolism patterns are present at preterm gestation, we analyzed SPA, SPH, and S1P levels in preterm non-labor (PTNL) tissues. Despite our analysis being limited to PTNL samples, SPA and SPH distribution mirrored those of TNL tissues. SPA was more abundant in the decidua parietalis than in the chorioamnion and myometrium (Fig. 6A), while SPH was similar between the decidua parietalis and myometrium and lowest in the chorioamnion (Fig. 6B). In contrast to the distribution pattern at term, S1P abundance was similar between the decidua parietalis and myometrium and lowest in the chorioamnion (Fig. 6C).

We next compared these metabolites between PTNL and TNL to evaluate gestational age-dependent differences. SPA and SPH abundances were similar between PTNL and TNL (Table S7) [23]. In contrast, S1P was significantly higher at TNL than at PTNL in the chorioamnion and myometrium (Table S7) [23], suggesting a gestational age-dependent increase in S1P.

Finally, we assessed S1P production and turnover by examining S1P:SPA and S1P:SPH ratios. At PTNL, the S1P:SPA ratio was higher in the myometrium than in the decidua parietalis (Fig. 6D), suggesting increased S1P turnover activity via the *de novo* pathway. No significant differences in the S1P:SPH ratio were observed between tissues at PTNL (Fig. 6E). When comparing the gestational stages, the S1P:SPH ratio was higher in the myometrium at TNL than at PTNL (Table S7) [23], while the S1P:SPA ratio showed no difference between the two gestational stages.

## Discussion

This study investigated the expression of S1P metabolic enzymes, S1P receptors, and sphingolipid abundance in the human chorioamnion, decidua parietalis, and myometrium during preterm and term gestation. SPHK1, the rate-limiting enzyme for extracellular S1P production, exhibited the highest abundance in the decidua parietalis at TNL, and its expression in the myometrium was higher during TL than TNL. Furthermore, S1P concentrations in the myometrium were significantly elevated at TL compared to TNL, indicating labor-dependent regulation of S1P in the tissue. Moreover, S1P levels were higher in the myometrium at TNL than at PTNL, suggesting that there is further gestational age-dependent regulation of S1P in the tissue. Finally, S1PR1-4 were most abundant in the myometrium, suggesting that this tissue is most responsive to S1P signaling.

Sphingolipid metabolites, including ceramide, sphingosine, and S1P, are known to be bioactive signaling molecules that regulate cell migration, differentiation, survival, and numerous other biological properties [27]. Although multiple sphingolipids have bioactive properties, S1P is the most potent and well-studied. Sphingolipid biosynthesis can occur via three distinct pathways, including the *de novo* pathway with sphinganine and ceramide as precursors, the sphingomyelinase pathway with sphingomyelins and ceramides as precursors, or the salvage pathway with sphingosine as the precursor [28, 29]. These pathways all share a final rate-limiting step involving the phosphorylation of sphingosine-by-sphingosine kinases (SPHK1 and SPHK2) to generate S1P [28]. Thus, SPHK expression has been used as a surrogate of S1P production, concentration, and activity. Studies in rats and sheep have shown that SPHK1 expression increases in the uterus, including the myometrium, as gestation progresses [30, 31]. Similarly, a human study has shown that SPHK1 protein and activity increase progressively in the decidua throughout pregnancy [19].

With respect to parturition, SPHK1 expression was found to be higher in the human amniotic membrane at postpartum than at TNL [32]. Similarly, our group previously showed that SPHK1 gene expression was significantly higher in the myometrium of patients at TL compared to those at TNL [11]. Our current study supports and expands on these findings by quantifying the sphingolipid metabolites rather than solely relying on SPHK1 as a surrogate of S1P production. This approach is more comprehensive, as S1P levels are influenced by additional factors such as the presence and activity of S1P degradation enzymes and cellular transport mechanisms.

We showed that *SPHK1* gene expression was higher in the myometrium at TL than at TNL, but this difference was not observed in the decidua. This spatial distinction suggests a specific association between S1P and the transition of the myometrium to a contractile state with the onset of labor, which is further supported by the higher abundance of S1P levels in the myometrium than in the decidua and chorioamnion at TL. These findings align with studies implicating S1P in preterm and term labor [18, 33-35]. One of the most compelling studies has shown that global inhibition of SPHK1/2, and therefore production of S1P, prevented inflammation-induced preterm labor in mice [20]. This was accompanied by reduced pro-inflammatory cytokines, suggesting that S1P plays a crucial role in triggering labor through inflammatory signaling pathways. Additional functional studies revealed that exogenous S1P induces key inflammatory markers in human and rat myometrial cells, including cyclooxygenase-2 (COX-2) and IL-8, both essential for parturition [11, 30, 36]. Beyond inflammation, S1P also promotes myometrial contractility through activation of the RhoA/Rho kinase pathway, a direct signaling cascade in labor contractions [37, 38]. RhoA/Rho kinase initiates contractions by inhibiting myosin light chain phosphatase (MLCP), thereby sustaining phosphorylation of MLC. Phosphorylated MLC enables actin-myosin cross-bridge formation that drives contractions [39]. These data suggest that elevated myometrial SPHK1 expression and S1P abundance at TL may facilitate both pro-inflammatory and pro-contractile signaling required for labor initiation. Additionally, accumulating evidence suggests that SPHK1 is hormonally regulated during pregnancy. Progesterone, a key hormone for pregnancy maintenance, promotes uterine quiescence, and its functional withdrawal near term permits myometrial activation leading to labor onset [40]. Consistent with this role, progesterone has been shown to increase SPHK1 expression in the rat myometrium, while progesterone withdrawal decreases SPHK1 and increases S1PL, the enzyme responsible for irreversible degradation of S1P [38]. Together, these findings suggest that progesterone-regulated SPHK1-mediated S1P production may contribute to labor onset by simultaneously activating pro-inflammatory and pro-contractile signaling mechanisms.

We further found that S1P abundance was higher in the chorioamnion and myometrium at TNL than at PTNL. This finding is consistent with previous work that reported S1P was higher in the chorioamnion post-labor than at TNL [32]. The S1P:SPA ratio was not altered across gestational age, suggesting stable *de novo* production of S1P during pregnancy. However, the higher S1P:SPH ratio in the myometrium at TNL than at PTNL suggests increased S1P production due to increased SPHK1/2 activity with gestational age.

The primary source of S1P production within the gestational tissues remains a subject of ongoing inquiry. While some studies suggest that S1P produced in the amniotic tissue is a significant contributor during labor [32], others have implicated the decidua [19]. Our sphingolipid metabolite profiling showed sphingomyelin, sphinganine, ceramide, and sphingosine levels were most abundant either in the decidua or equally abundant in the decidua and myometrium. We found consistently low levels of all sphingolipid metabolites, including *SPHK1*, in the chorioamnion compared to the maternal tissue. Instead, our data suggests that the decidua may play a substantial role in sphingolipid production, with elevated levels of sphingosine acting as a precursor for S1P. Our analysis, using matched samples, further demonstrated that *SPHK1* expression was higher in the decidua compared to the chorioamnion or myometrium at TNL, a crucial period when the decidua is priming for labor onset. Additionally, SM C16:0 was the most prominent sphingomyelin, while ceramide-C16:0 was the most prominent ceramide across all three gestational tissues at term pregnancy. Notably, ceramide-C16:0 was more abundant in the decidua than the chorioamnion at TNL, further illustrating the role of the decidua in regulating key sphingolipid metabolites involved in labor initiation. A notable exception was SM C16:0 and SM C18:0, which were found to be more abundant in the myometrium than in the decidua, particularly at TNL. While little is known about the function of sphingomyelin within tissues during pregnancy, decreased plasma levels of SM C16:0 and SM C18:0 during the first trimester have been associated with preeclampsia [25]. Research has also found increased plasma concentrations of ceramide-C16:0, a metabolite of SM C16:0, in patients experiencing early preterm labor compared to controls [41]. These findings suggest a potential role for these sphingolipid subclasses in gestational pathology or as biomarkers of labor, highlighting the necessity for further research on their functions in gestational tissue during labor.

S1P breakdown is regulated by the enzymes S1PP1 and S1PL. We found that the decidua and myometrium exhibited markedly higher levels of *S1PP1* than the chorioamnion at TNL. However, *S1PL* expression was more pronounced in the myometrium than in the decidua and chorioamnion at TNL. At TL, we did not observe any significant difference in either the S1P biosynthesis or degradation enzymes across the tissues, suggesting that most of the S1P enzymatic activity occurs before labor onset to accumulate sufficient S1P levels necessary for signaling during parturition. The role of SPHK2 in pregnancy remains to be studied. However, we noted higher *SPHK2* expression in the myometrium than in the chorioamnion and decidua at TNL, suggesting that SPHK2 may also contribute to the regulation of myometrial contractility.

Extracellular S1P exerts its biological effects by binding to one of its five G protein-coupled receptors, S1PR1-5, that initiate a diverse range of downstream signaling cascades [42]. While all five S1P receptors bind S1P with nanomolar affinity, they differ in downstream G-protein coupling and cellular responses. We previously reported that S1P mediates its pro-inflammatory effects in human myometrial cells via S1PR3 [11], coinciding with findings that S1PR3 expression increased with gestation in human decidua [19]. Here, we found that *S1PR1-3* were more abundant in the myometrium than in the chorioamnion and decidua at term gestation, suggesting that the myometrium is the most responsive of these tissues to S1P signaling. There were no significant differences in *S1PR1-3* in the decidua or *S1PR1-2* expression in the myometrium between TL and TNL. An unexpected finding was decreased *S1PR3* expression in the myometrium at TL than TNL. The reduced receptor expression could be attributed to a negative feedback loop triggered by increased S1P signaling during the inflammatory process of labor, serving to maintain cellular homeostasis. It is crucial to note that S1P receptor signaling exhibits tissue- and cell-type-specificity, with the unique ability to elicit diverse responses depending on the environmental stimuli. Indeed, this is observed with S1PR1 expression, which increases only in chronic but not acute inflammation during inflammatory bowel disease [43]. Therefore, the complex interplay of labor and S1P signaling mechanisms may lead to differential regulation of the S1P receptors, including S1PR3.

While our study offers valuable insights, it is important to acknowledge several limitations. First, the small sample size may have limited our ability to detect differences, potentially leading to false negative findings for some of the metabolites. Second, we did not perform a priori power calculation as there were no available data on the sphingolipid metabolite concentrations in human gestational tissues. Nonetheless, the data presented here can now be used to inform power estimates and guide the design of larger studies in the future. We were also unable to include preterm labor samples due to their limited availability. Additionally, our analyses were performed on whole-tissue samples rather than at the single-cell level, which may mask cell-specific differences in sphingolipid signaling. Future investigations utilizing single-cell approaches could provide a more detailed understanding of the distinct roles that sphingolipids play within individual cell types in gestational tissues. S1P metabolic enzymes and receptor expression were assessed only at the mRNA level, which may not reflect protein abundance or activity. Finally, our data demonstrates an association, not causation, between S1P concentrations in the human uterus and labor. These findings should therefore be interpreted with caution. Future mechanistic studies are needed to define causal pathways and translate these observations into clinically actionable insights. Despite these constraints, our study is strengthened by an integrated analysis of S1P metabolic pathway gene expression and quantitative lipidomic profiling of sphingolipid metabolites in gestational tissues at three time points of gestation. Furthermore, we conducted our analyses using matched samples from the chorioamnion, decidua, and myometrium, which enhances the robustness and relevance of our findings.

Effectively managing labor complications, including preterm labor and labor dystocia, remains a challenge because current medications have off-target effects that result in adverse maternal and fetal outcomes [44]. S1P signaling is known to be mediated through its binding to and activation of one of five S1P receptors (S1PR1-5). Each of the five receptors has distinct tissue, cellular, and temporal expression patterns [45, 46], and S1PR3 has been identified as the predominant receptor modulating S1P effects in the human myometrium [11]. This receptor tissue-specificity offers a potential approach to minimize the systemic maternal side effects associated with the current therapies. Our analysis additionally revealed a striking tissue-specific difference in the abundance of S1P metabolites and expression of associated enzymes and receptors. This pronounced partitioning adds additional support for targeting S1P signaling in preterm labor prevention or treatment and potentially bypassing fetal tissue exposure and minimizing the adverse fetal effects associated with current systemic therapies.

**Figure 7:**
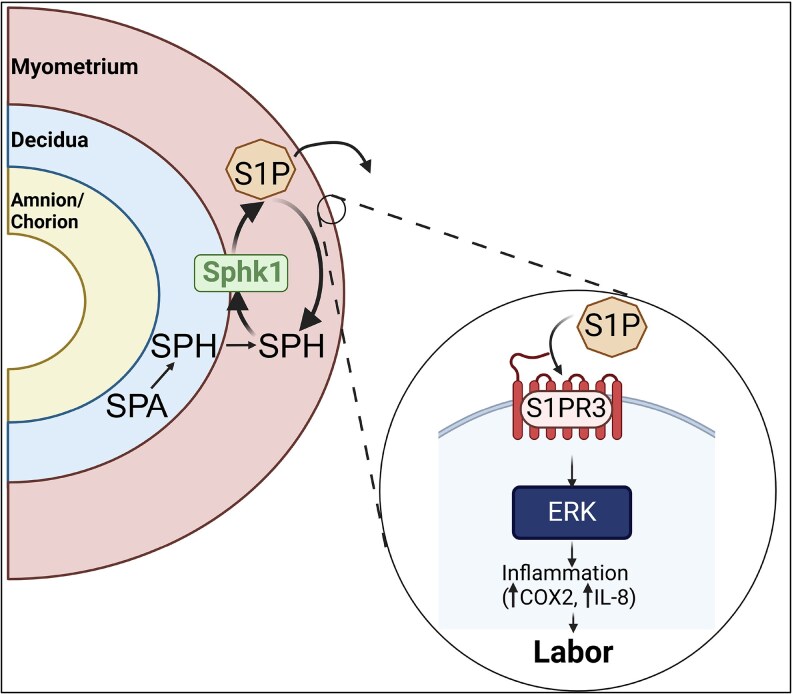
A proposed pathway for S1P metabolism and signaling in human gestational tissue. Sphingosine, primarily in the decidua and myometrium, is converted to S1P via SPHK1. S1P predominantly acts on the myometrium via S1PR3 to induce COX2 and IL8 expression, promoting inflammatory processes associated with labor onset.

In conclusion, our study provides the first longitudinal, multi-tissue atlas of sphingolipid dynamics in the human gestational tissues. We demonstrate that S1P metabolism is differentially regulated across gestational tissues during pregnancy and labor. Notably, we showed that S1P production and signaling are highly modulated in the myometrium during labor (Fig. 7). This tissue-specific expression pattern highlights S1P signaling as a precision therapeutic target for labor-related complications, with potentially reduced off-target fetal side effects. This work lays the foundation for future studies to investigate the potential efficacy and safety of S1P-based therapies in gestational models for modulating the myometrium during labor.

## Data Availability

Original data generated and analyzed during this study are included in this published article or in the repositories listed in References.

## References

[1] Osman I, Young A, Ledingham MA, et al Leukocyte density and pro-inflammatory cytokine expression in human fetal membranes, decidua, cervix and myometrium before and during labour at term. Mol Hum Reprod. 2003;9(1):41‐45. 12529419 10.1093/molehr/gag001

[2] Smith R. Mechanisms of disease parturition. N Engl J Med. 2007;356(3):271‐283. 17229954 10.1056/NEJMra061360

[3] Romero R, Dey SK, Fisher SJ. Preterm labor: one syndrome, many causes. Science (1979). 2014;345(6198):760‐765. 10.1126/science.1251816 PMC4191866 25124429

[4] Green ES, Arck PC. Pathogenesis of preterm birth: bidirectional inflammation in mother and fetus. Semin Immunopathol. 2020;42(4):413‐429. 32894326 10.1007/s00281-020-00807-y PMC7508962

[5] Galal M, Symonds I, Murray H, Petraglia F, Smith R. Postterm pregnancy. Facts Views Vis Obgyn. 2012;4(3):175‐187. 24753906 PMC3991404

[6] Kissler K, Hurt KJ. The pathophysiology of labor dystocia: theme with variations. Reprod Sci. 2023;30(3):729‐742. 35817950 10.1007/s43032-022-01018-6 PMC10388369

[7] March of Dimes . Long-term health effects of preterm birth. Accessed November 20, 2024. https://www.marchofdimes.org/find-support/topics/birth/long-term-health-effects-preterm-birth

[8] Knigin D, Ezra Y, Ben-David A, Elami-Suzin M. The continuum of a prolonged labor and a second stage cesarean delivery. J Matern Fetal Neonatal Med. 2022;35(25):6425‐6429. 34030598 10.1080/14767058.2021.1914577

[9] Barber EL, Lundsberg LS, Belanger K, Pettker CM, Funai EF, Illuzzi JL. Indications contributing to the increasing cesarean delivery rate. Obstet Gynecol. 2011;118(1):29‐38. 21646928 10.1097/AOG.0b013e31821e5f65 PMC3751192

[10] Gifford DS, Morton SC, Fiske M, Keesey J, Keeler E, Kahn KL. Lack of progress in labor as a reason for cesarean. Obstet Gynecol. 2000;95(4):589‐595. 10725495 10.1016/s0029-7844(99)00575-x

[11] Saurabh K, Mbadhi MN, Prifti KK, Martin KT, Frolova AI. Sphingosine 1-phosphate activates S1PR3 to induce a proinflammatory phenotype in human myometrial cells. Endocrinology. 2023;164(6):bqad066. 37120767 10.1210/endocr/bqad066 PMC10201982

[12] Maceyka M, Milstien S, Spiegel S. Sphingosine kinases, sphingosine-1-phosphate and sphingolipidomics. Prostaglandins Other Lipid Mediat. 2005;77(1-4):15‐22. 16099387 10.1016/j.prostaglandins.2004.09.010

[13] Johnson KR, Becker KP, Facchinetti MM, Hannun YA, Obeid LM. PKC-dependent activation of sphingosine kinase 1 and translocation to the plasma membrane: extracellular release of sphingosine-1-phosphate induced by phorbol 12-myristate 13-acetate (PMA). J Biol Chem. 2002;277(38): 35257‐35262. 12124383 10.1074/jbc.M203033200

[14] Diaz Escarcega R, McCullough LD, Tsvetkov AS. The functional role of sphingosine kinase 2. Front Mol Biosci. 2021;8:683767. 34055895 10.3389/fmolb.2021.683767 PMC8160245

[15] Song DD, Zhou JH, Sheng R. Regulation and function of sphingosine kinase 2 in diseases. Histol Histopathol. 2018;33(5):433‐445. 29057430 10.14670/HH-11-939

[16] Strub GM, Paillard M, Liang J, et al Sphingosine-1-phosphate produced by sphingosine kinase 2 in mitochondria interacts with prohibitin 2 to regulate complex IV assembly and respiration. FASEB J. 2011;25(2):600‐612. 20959514 10.1096/fj.10-167502 PMC3023391

[17] Hannun YA, Luberto C, Argraves KM. Enzymes of sphingolipid metabolism: from modular to integrative signaling. Biochemistry. 2001;40(16):4893‐4903. 11305904 10.1021/bi002836k

[18] Mizugishi K, Li C, Olivera A, et al Maternal disturbance in activated sphingolipid metabolism causes pregnancy loss in mice. J Clin Invest. 2007;117(10):2993‐3006. 17885683 10.1172/JCI30674 PMC1978422

[19] Yamamoto Y, Olson DM, Van Bennekom M, Brindley DN, Hemmings DG. Increased expression of enzymes for sphingosine 1-phosphate turnover and signaling in human decidua during late pregnancy. Biol Reprod. 2010;82(3):628‐635. 20007411 10.1095/biolreprod.109.081497

[20] Vyas V, Ashby CR, Olgun NS, et al Inhibition of sphingosine kinase prevents lipopolysaccharide-induced preterm birth and suppresses proinflammatory responses in a murine model. Am J Pathol. 2015;185(3):862‐869. 25579843 10.1016/j.ajpath.2014.10.026 PMC4348465

[21] Giusto K, Ashby CR. Investigating the Et-1/SphK/S1P pathway as a novel approach for the prevention of inflammation-induced preterm birth. Curr Pharm Des. 2018;24(9):983‐988. 29384055 10.2174/1381612824666180130122739

[22] Farine T, Parsons M, Lye S, Shynlova O. Isolation of primary human decidual cells from the fetal membranes of term placentae. J Vis Exp. 2018;134:57443. 10.3791/57443 PMC6101051 29757275

[23] Mbadhi MN, Fujiwara H, Gill R, et al Supplemental data: the sphingosine-1-phosphate pathway is differentially activated in human gestational tissues. Washington University in St. Louis. 2026. 10.17632/nxyvc4hjfc PMC13080204 41994486

[24] Fan M, Sidhu R, Fujiwara H, et al Identification of Niemann-Pick C1 disease biomarkers through sphingolipid profiling. J Lipid Res. 2013;54(10):2800‐2814. 23881911 10.1194/jlr.M040618 PMC3770093

[25] Dobierzewska A, Soman S, Illanes SE, Morris AJ. Plasma cross-gestational sphingolipidomic analyses reveal potential first trimester biomarkers of preeclampsia. PLoS One. 2017;12(4):e0175118. 28384202 10.1371/journal.pone.0175118 PMC5383057

[26] Hammad SM, Pierce JS, Soodavar F, et al Blood sphingolipidomics in healthy humans: impact of sample collection methodology. J Lipid Res. 2010;51(10):3074‐3087. 20660127 10.1194/jlr.D008532 PMC2936747

[27] Bartke N, Hannun YA. Bioactive sphingolipids: metabolism and function. J Lipid Res. 2009;50(Suppl.): S91‐S96. 19017611 10.1194/jlr.R800080-JLR200 PMC2674734

[28] Gault CR, Obeid LM, Hannun YA. An overview of sphingolipid metabolism: from synthesis to breakdown. Adv Exp Med Biol 2010; 688:1‐23. 20919643 10.1007/978-1-4419-6741-1_1 PMC3069696

[29] Merrill AH. De novo sphingolipid biosynthesis: a necessary, but dangerous, pathway. J Biol Chem. 2002;277(29): 25843‐25846. 12011104 10.1074/jbc.R200009200

[30] Serrano-Sanchez M, Tanfin Z, Leiber D. Signaling pathways involved in sphingosine kinase activation and sphingosine-1-phosphate release in rat myometrium in late pregnancy: role in the induction of cyclooxygenase 2. Endocrinology. 2008;149(9):4669‐4679. 18723875 10.1210/en.2007-1756

[31] Dunlap KA, Kwak H Il, et al The sphingosine 1-phosphate (S1P) signaling pathway is regulated during pregnancy in sheep. Biol Reprod. 2010;82(5):876‐887. 20107206 10.1095/biolreprod.109.081604 PMC2857631

[32] Erkhembaatar LO, Kotani T, Sumigama S, et al Increased expression of sphingosine kinase in the amnion during labor. Placenta. 2013;34(4):353‐359. 23462226 10.1016/j.placenta.2013.01.014

[33] Enthoven LF, Shi Y, Fay E, et al Effects of pregnancy on plasma sphingolipids using a metabolomic and quantitative analysis approach. Metabolites. 2023;13(9): 1026. 37755306 10.3390/metabo13091026 PMC10534641

[34] Lantzanaki M, Vavilis T, Harizopoulou VC, Bili H, Goulis DG, Vavilis D. Ceramides during pregnancy and obstetrical adverse outcomes. Metabolites. 2023;13(11):1136. 37999232 10.3390/metabo13111136 PMC10673483

[35] Fakhr Y, Brindley DN, Hemmings DG. Physiological and pathological functions of sphingolipids in pregnancy. Cell Signal. 2021; 85:110041. 33991614 10.1016/j.cellsig.2021.110041

[36] Erkinheimo TL, Saukkonen K, Narko K, Jalkanen J, Ylikorkala O, Ristimäki A. Expression of cyclooxygenase-2 and prostanoid receptors by human myometrium*. J Clin Endocrinol Metab. 2000;85(9): 3468‐3475. 10999850 10.1210/jcem.85.9.6809

[37] Leiber D, Banno Y, Tanfin Z. Exogenous sphingosine 1-phosphate and sphingosine kinase activated by endothelin-1 induced myometrial contraction through differential mechanisms. Am J Physiol Cell Physiol. 2007;292(1):240‐250. 10.1152/ajpcell.00023.2006 16956968

[38] Jeng YJ, Suarez VR, Izban MG, Wang HQ, Soloff MS. Progesterone-induced sphingosine kinase-1 expression in the rat uterus during pregnancy and signaling consequences. Am J Physiol Endo-crinol Metab. 2007;292(4):1110‐1121. 10.1152/ajpendo.00373.2006 17164439

[39] Khromov A, Choudhury N, Stevenson AS, Somiyo A V., Eto M. Phosphorylation-dependent autoinhibition of myosin light chain phosphatase accounts for Ca2+ sensitization force of smooth muscle contraction. J Biol Chem. 2009;284(32):21569‐21579. 19531490 10.1074/jbc.M109.019729 PMC2755881

[40] Astle S, Slater DM, Thornton S. The involvement of progesterone in the onset of human labour. Eur J Obstet Gynecol Reprod Biol. 2003;108(2):177‐181. 12781407 10.1016/s0301-2115(02)00422-0

[41] Laudanski P, Charkiewicz K, Kisielewski R, et al Plasma C16-Cer levels are increased in patients with preterm labor. Prostaglandins Other Lipid Mediat. 2016;123:40‐45. 27184754 10.1016/j.prostaglandins.2016.04.005

[42] Takuwa Y. Roles of sphingosine-1-phosphate signaling in angiogenesis. World J Biol Chem. 2010;1(10), 298. 21537463 10.4331/wjbc.v1.i10.298 PMC3083935

[43] Karuppuchamy T, Behrens EH, González-Cabrera P, et al Sphingosine-1-phosphate receptor-1 (S1P 1) is expressed by lymphocytes, dendritic cells, and endothelium and modulated during inflammatory bowel disease. Mucosal Immunol. 2017;10(1), 162‐171. 27049060 10.1038/mi.2016.35 PMC5053832

[44] Lamont RF, Jørgensen JS. Safety and efficacy of tocolytics for the treatment of spontaneous preterm labour. Curr Pharm Des. 2019;25(5):577‐592. 30931850 10.2174/1381612825666190329124214

[45] Blaho VA, Hla T. An update on the biology of sphingosine 1-phosphate receptors. J Lipid Res. 2014;55(8):1596‐1608. 24459205 10.1194/jlr.R046300 PMC4109755

[46] Bravo GÁ, Cedeño RR, Casadevall MP, Ramió-Torrentà L. Sphingosine-1-phosphate (S1P) and S1P signaling pathway modulators, from current insights to future perspectives. Cells. 2022;11(13): 2058. 35805142 10.3390/cells11132058 PMC9265592

